# Modification of the Tumor Microenvironment in KRAS or c-MYC-Induced Ovarian Cancer-Associated Peritonitis

**DOI:** 10.1371/journal.pone.0160330

**Published:** 2016-08-02

**Authors:** Mitsuyo Yoshida, Ayumi Taguchi, Kei Kawana, Katsuyuki Adachi, Akira Kawata, Juri Ogishima, Hiroe Nakamura, Asaha Fujimoto, Masakazu Sato, Tomoko Inoue, Haruka Nishida, Hitomi Furuya, Kensuke Tomio, Takahide Arimoto, Kaori Koga, Osamu Wada-Hiraike, Katsutoshi Oda, Takeshi Nagamatsu, Tohru Kiyono, Yutaka Osuga, Tomoyuki Fujii

**Affiliations:** 1 Department of Obstetrics and Gynecology, Graduate School of Medicine, The University of Tokyo, 7-3-1 Hongo, Bunkyo-ku, Tokyo 113–8655, Japan; 2 Division of Virology, National Cancer Center Research Institute, 5-1-1 Tsukiji, Chuo-ku, Tokyo, 104–0045, Japan; Cincinnati Children's Hospital Medical Center, UNITED STATES

## Abstract

The most common properties of oncogenes are cell proliferation and the prevention of apoptosis in malignant cells, which, as a consequence, induce tumor formation and dissemination. However, the effects of oncogenes on the tumor microenvironment (TME) have not yet been examined in detail. The accumulation of ascites accompanied by chronic inflammation and elevated concentrations of VEGF is a hallmark of the progression of ovarian cancer. We herein demonstrated the mechanisms by which oncogenes contribute to modulating the ovarian cancer microenvironment. c-MYC and KRAS were transduced into the mouse ovarian cancer cell line ID8. ID8, ID8-c-MYC, or ID8-KRAS cells were then injected into the peritoneal cavities of C57/BL6 mice and the production of ascites was assessed. ID8-c-MYC and ID8-KRAS both markedly accelerated ovarian cancer progression *in vivo*, whereas no significant differences were observed in proliferative activity *in vitro*. ID8-KRAS in particular induced the production of ascites, which accumulated between approximately two to three weeks after the injection, more rapidly than ID8 and ID8-c-MYC (between nine and ten weeks and between six and seven weeks, respectively). VEGF concentrations in ascites significantly increased in c-MYC-induced ovarian cancer, whereas the concentrations of inflammatory cytokines in ascites were significantly high in KRAS-induced ovarian cancer and were accompanied by an increased number of neutrophils in ascites. A cytokine array revealed that KRAS markedly induced the expression of granulocyte macrophage colony-stimulating factor (GM-CSF) in ID8 cells. These results suggest that oncogenes promote cancer progression by modulating the TME in favor of cancer progression.

## Introduction

Ovarian cancer is effectively treated with platinum-based chemotherapies. However, approximately 15–20% of ovarian cancer cases are resistant to this treatment [1]. Although surgery followed by paclitaxel and carboplatin (TC) therapy is the standard initial treatment for ovarian cancer, 60% or more of patients need second-line chemotherapy. A treatment regimen for second-line chemotherapy against recurrence after TC therapy has yet to be established [2]. Furthermore, therapies that target the tumor microenvironment (TME), including angiogenesis and inflammation, in ovarian cancer characterized by peritoneal dissemination accompanied by massive ascites are novel strategies [3]. Adjuvant therapy may be effective for each case by identifying a tumor-promoting factor (inflammation, angiogenesis, or immunosuppression).

The accumulation of ascites accompanied by chronic inflammation and increased concentrations of VEGF is a hallmark of the progression of ovarian cancer. Regarding inflammation, increasing evidence has revealed that the activation of NF-κB in cancer cells is associated with the more rapid progression of ovarian cancer [4], leading to changes in the TME, such as the activation of macrophages. VEGF itself or VEGF-induced angiogenesis is crucial for disseminated ovarian cancer with ascites. Therefore, the control of inflammatory signaling and targeting of VEGF pathways have recently been attracting attention due to their potential as ideal strategies for ovarian cancer [5].

The most common properties of oncogenes are cell proliferation and prevention of apoptosis in malignant cells, which, as a consequence, induce tumor formation and dissemination. However, few studies have examined the relationship between oncogenes and modifications to the TME.

In ovarian cancers, Ronald L et al. revealed that the co-existence of an ARID1A-PIK3CA mutation promoted ovarian clear cell tumorigenesis by increasing the expression of IL-6 [6]. IL-6 is a central cytokine that is mainly produced by TME components such as cancer-associated fibroblasts (CAF) and tumor-associated macrophages (TAM) [7. 8], and contributes to cancer progression by stimulating cancer cells and modulating the TME. A previous study indicated that the characteristics of tumor-infiltrating lymphocytes (TIL) are influenced by the disruption of BRCA1, which is a well-known tumor suppressor gene of ovarian cancer [9]. These findings suggest that the activation of oncogenes or disruption of tumor suppressor genes is involved in modulations to the TME.

Based on the findings described above, we hypothesized that oncogenes modulate the TME in favor of cancer progression. In the present study, which focused on angiogenesis and inflammation, we established oncogene-transduced mouse ovarian cancer cell lines by transducing c-MYC and KRAS into the mouse ovarian epithelial immortalized cell line, ID8, which has been established from C57BL/6 mice. We then investigated TME modifications caused by oncogenes using a mouse *in vivo* model of the disseminated ovarian cancer cell lines.

## Materials and Methods

### Establishment of oncogene-transduced ID8

ID8 cells were kindly gifted by Dr. Kathy Roby, Department of Anatomy and Cell Biology, University of Kansas Medical Center. A mutant form of human MYC (MYC^T58A^) [10] was recombined into CSII(ins)-CMV-RfA in order to generate CSII(ins)-CMV-MYC^T58A^. CSII(ins)-CMV-RfA was constructed by inserting the 1.2-kb chicken b-globin insulator sequence (cHS4) into the *Bst*EII site of the 3’LTR of CSII-CMV-RfA. ID8-MYC cells were established by transducing the CSII(ins)-CMV-MYC^T58A^ VSV-G pseudotyped lentivirus at a multiplicity of infection of 10 as described previously, and then cultured in DMEM (Gibco, NY, USA) containing 10% FBS, 100 U/ml penicillin, 0.1 mg/ml streptomycin, and 0.25 g/ml amphotericin B [10, 11]. An oncogenic mutant form of human KRAS (KRAS^G12V^) was recombined into pDEST-CLXSN to generate pCLXSN-KRAS^G12V^. Packaging of the retroviruses were as described previously. ID8-KRAS cells were established by infection of the LXSN-KRAS^G12V^ virus at a multiplicity of infection of 1, followed by G418 selection at a concentration of 800 μg/ml for a week.

### Mouse model

ID8, ID8-c-MYC, and ID8-KRAS cells were cultured in Dulbecco’s Modified Eagle Medium (DMEM) (Gibco, NY, USA) containing 10% FBS, 100 U/ml penicillin, 0.1 mg/ml streptomycin, and 0.25 g/ml amphotericin B. C57BL/6J mice were chosen because ID8 was established from C57BL/6J mice [12]. Mice were purchased from Japan SLC, Inc. ID8, ID8-KRAS, and ID8-c-MYC cells (2× 10^6^) suspended in 1000 μl of DMEM were injected into the peritoneal cavities of 8-week-old female mice under anesthesia by isoflurane. ID8-KRAS mice were monitored every other day, and ID8 and ID8-c-MYC mice were monitored twice a week. Mice were sacrificed by isoflurane when their body weight (BW) reach 23 g after the inoculation. At the time of sacrifice, BW and ascites weights were assessed. Mice were sacrificed to minimize suffering when moribund behaviors were observed. Total number of mice used in this study was 82 as following: for observation of phenotype and sample collection, ID8 mice group: n = 10, ID8-c-MYC mice group: n = 10, ID8-KRAS mice group: n = 10, for time course analysis: n = 20, for Giemsa assay: n = 16 (n = 4 per group), for the assessment of leukocytes proportion by FACS: control mice: n = 4, ID8 mice: n = 6, and ID8-KRAS mice: n = 6. Animal studies were approved by the University of Tokyo Animal Committee.

### Cell proliferation assay

ID8, ID8-c-MYC, and ID8-KRAS cells were plated on 96-well culture plates at a concentration of 5×10^4^ cells/ml and cultured for 48 h. Cell proliferation assay was performed as previously reported [13] and assessed by the Cell Counting kit-8 (CCK-8) assay (DOJINDO, Osaka, Japan) according to the manufacturer’s instructions: absorbance at 490 nm was measured using a micro-plate reader (BioTek, USA) and the mean ratio of absorbance was calculated.

### Immunohistochemistry (IHC)

Ki67 and CD-31 IHC of tumor sections was performed as previously described [14]. Paraffin sections (4-μm-thick) of the biggest tumor sections from ID8, ID8-c-MYC, and ID8-KRAS mice (sections were taken when BW exceeded 23 g) were dewaxed in xylene and rehydrated through graded ethanol to water. Antigens were retrieved by boiling in 10 mM citrate buffer (pH 6.0) for 30 min. The cooled sections were incubated in DAKO REAL Peroxidase-Blocking solution (DAKO, Carpinteria, CA, USA) for 10 min to quench endogenous peroxidase. Sections were incubated in DAKO Protein Blocking solution (DAKO) at room temperature for 10 min to block non-specific binding. Sections were then stained for Ki67 using rabbit monoclonal antibody against mouse Ki67(1:100; Spring Bioscience, CA, USA), CD-31 using a rat monoclonal antibody against mouse CD-31 (ab56299, Abcam, Tokyo, Japan, 1:100 dilution).

### Isolation of leukocytes and Giemsa staining

Peritoneal cells were obtained from ID8-, ID8-c-MYC-, or ID8-KRAS-induced ascites. Blood cells were also collected from each group. Peritoneal and blood leukocytes were isolated from the collected peritoneal cells using a magnetic cell sorting kit (MACS; Miltenyi Biotec K.K. Cologne, Germany) according to the manufacturer’s instructions. The magnetically labeled CD45-positive cells were eluted as a positively selected cell fraction. Peritoneal leukocytes were stained with Diff Quick® (LT-SYS®, E. Lehmann GmbH, and Berlin).

### Flow cytometry

Samples were suspended in 500 μl of PBS/1% BSA and analyzed by flow cytometry (FACS Caliber; Becton Dickinson, Mountain View, CA, USA). Matched isotype antibodies were used as controls. Neutrophils were stained with the following antibodies: FITC-anti-mouse CD45 (Beckman Coulter, clone: B3821F4A / N901 / UCHT1), APC-anti-mouse Ly6G (eBioscience, clone: RB6-8C5), FITC-mouse IgG1, κ isotype(Biolegend, clone: MOPC-21), and APC-mouse IgG1, κ isotype (eBioscience, clone P3.6.2.8.1).

### RT-quantitative PCR (RT-qPCR)

RT-qPCR was performed as previously reported [15]. Total RNA was extracted from cultured ID8, ID8-c-MYC, and ID8-KRAS cells using a Favorgen extraction RNA kit (Tokyo, Japan, Chiyoda Science Co.), followed by reverse transcription. cDNA was amplified for 40 cycles in a Light Cycler 480 (Roche, Basel, Switzerland) using SYBR green I (Applied Biosystems). The primer pairs used were as follows: mouse peptidylprolyl isomerase A (PPIA), 5`-CGCGTCTCCTTCGAGCTGTTTG-3’ and 5`-TGTAAAGTCACCACCCTGGCACAT-3’; mouse Granulocyte Macrophage Colony-stimulating Factor (GM-CSF), 5`- GCCATCAAAGAAGCCCTGAA-3’ and 5`- GCGGGTCTGCACACATGTTA-3’. The PCR conditions used for PPIA were as follows: 35 cycles at 95°C for 10 s, 64°C for 10 s and 72°C for 6 s; and for GM-CSF, 40 cycles at 95°C for 10 s, 60°C for 10 s and 72°C for 6 s. The expression of GM-CSF was normalized using PPIA mRNA as the internal standard.

### Measurement of VEGF, inflammatory cytokines, and GM-CSF concentrations

VEGF, inflammatory cytokines (IL-6, IL-1β, and TNF-α), and GM-CSF concentrations were measured in ascites or cultured media using a specific ELISA kit (Quantikine; R&D Systems, Minneapolis, MN, USA), according to the manufacturer’s protocol.

### Statistical analysis

Data are presented as means ± SEM. Statistical analyses were conducted by the Student’s *t-*test or Dunnett’s analysis using JMP11. A value of *P* < 0.05 was considered significant. Asterisks indicate comparisons that were significantly different (*P* < 0.05).

## Results

### Oncogenes enhanced the production of ascites

**Fig 1:**
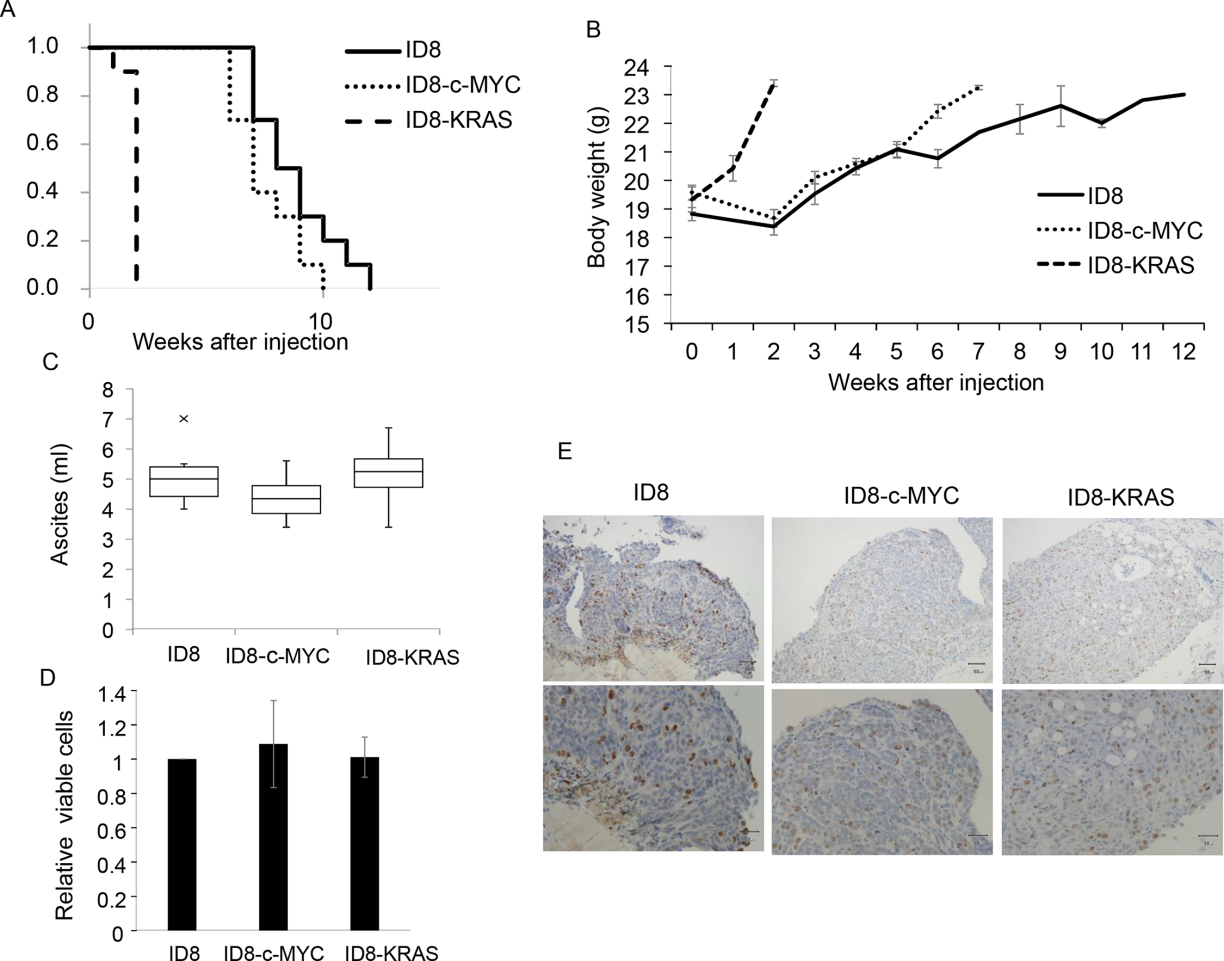
*In vivo* and *in vitro* characteristics of oncogene-transduced ID8 cells. (A, B, C) ID8, ID8-c-MYC, or ID8-KRAS cells (2 × 10^6^) were intra-peritoneal injected into C57/BL6 mice (ID8 group: n = 10, ID8-c-MYC group: n = 10, ID8-KRAS group: n = 10). ID8-KRAS mice were monitored every other day, and ID8 and ID8-c-MYC mice were monitored twice a week. Mice were sacrificed when the body weight reached 23 g. (A) The graph demonstrates Kaplan-Meier curve. The end point was when mice BW reached 23 g in each group. (B) The graph demonstrates that transition of BW in each group when mice BW reached. (C) The graph demonstrates the amount of ascites when BW in each group reached 23 g. Ascites weights were assessed using the following formula: (ascites weight) = (BW with ascites)—(BW without ascites). (D) ID8, ID8-c-MYC and ID8-KRAS cells were plated on 96-well culture plates at aconcentration of 5×10^4^ cells/ml and cultured for 48 h. The proliferation of ID8, ID8-c-MYC, and ID8-KRAS cells was assessed by cell proliferation assay. Each experiment was performed in triplicate and repeated 3 times. The average of three trials is shown. Error bars represent the mean ± SEM. (E) ID8, ID8-c-MYC, or ID8-KRAS cells (2 × 10^6^) were intra-peritoneal injected into C57/BL6 mice. Mice were sacrificed when the body weight reached 23 g. Disseminations were obtained from mice when sacrificed. Proliferation of tumor cells of each dissemination was assessed using Ki67 immunohistochemistry (IHC). Scale bars represent 50 μm and 30 μm at low and high magnification, respectively.

ID8, D8-c-MYC, or ID8-KRAS cells (2 × 10^6^) were injected into the peritoneal cavities of C57/BL6 mice (ID8 group: n = 10, ID8-c-MYC group: n = 10, ID8-KRAS group: n = 10). Mice were sacrificed when BW exceeded 23 g (the time when a moderate amount of ascites had accumulated and an appropriate point to be sacrificed). At that point, the production of ascites and formation of dissemination were observed in all groups. KRAS and c-MYC both accelerated the production of ascites, as shown in Fig 1A and 1B. As shown in Fig 1C, the amount of accumulated ascites when BW reached 23 g was almost 5 ml in all groups and the exaggeration of ovarian cancer progression was assumed to be similar in all groups at the time of sacrifice. ID8-KRAS in particular induced the production of ascites, in which ascites accumulated between approximately two to three weeks after the injection, more rapidly than ID8 and ID8-c-MYC (between nine and ten weeks and between six and seven weeks, respectively). To know whether there was difference in the proliferative ability between these cells, we conducted in vitro cell proliferation assay of each cell line, which revealed that there was no significant difference in proliferative activity among each cell line (Fig 1D). We also conducted in vivo assessment by Ki67 staining of disseminations of each group (Fig 1E). Upper (low-power field) and lower (high-power field) panels showed there was no obvious difference in the Ki67-positivity and invasive area of Ki67-positive cells between each group.

### c-MYC significantly increased VEGF levels in ascites

**Fig 2:**
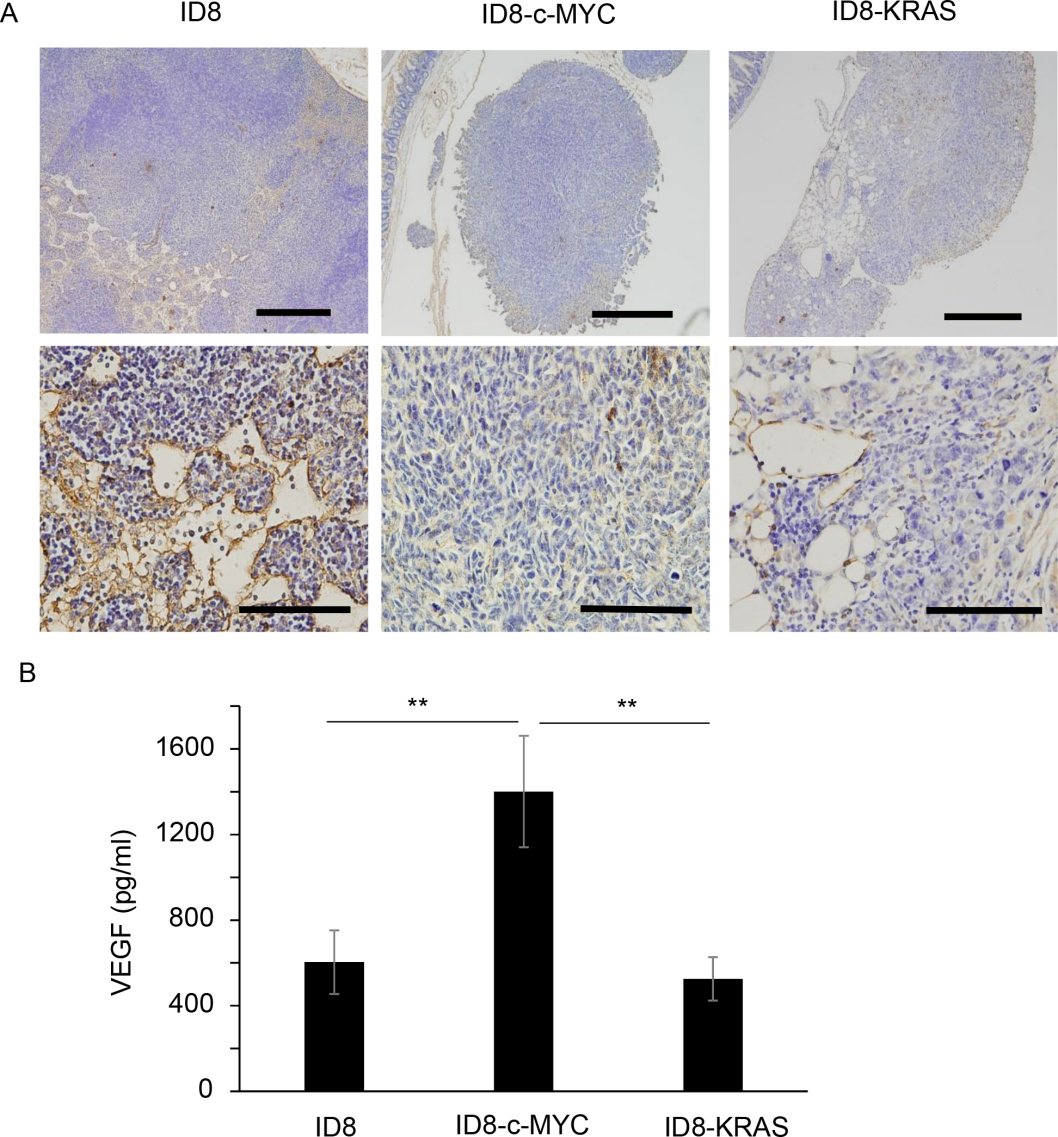
Tumor vascularization and VEGF in ascites. **(A)** ID8, ID8-c-MYC, or ID8-KRAS cells (2 × 10^6^) were intra-peritoneal injected into C57/BL6 mice. Mice were sacrificed when the body weight reached 23 g. Disseminations were obtained from mice when sacrificed. Tumor vascularization of each dissemination was assessed using CD-31 immunohistochemistry (IHC). Scale bars represent 200 μm and 50 μm at low and high magnification, respectively. (B) ID8, ID8-c-MYC, or ID8-KRAS cells (2 × 10^6^) were intra-peritoneal injected into C57/BL6 mice. Mice were sacrificed when the body weight reached 23 g and ascites were collected (ID8, ID8-c-MYC, and ID8-KRAS: n = 10). VEGF concentration of ascites were assessed with specific ELISA. Error bars represent the mean ± SEM. A statistical analysis was performed with the Student’s t-test (**P ≤ 0.01).

In order to investigate angiogenesis in each tumor, the number of microvessels was assessed using CD-31 IHC. However, since the size of dissemination was very small, few microvessels were detected by IHC, as shown in Fig 2A, and no significant differences were observed among the three groups.

Although the countable number of microvessels was not significantly different among these three groups, a significant difference was noted in VEGF concentrations in ascites; VEGF levels in ascites were markedly higher in ID8-c-MYC mice than in ID8 or ID8-KRAS mice (Fig 2B).

### Inflammatory cytokine concentrations were higher in KRAS-induced ascites

**Fig 3:**
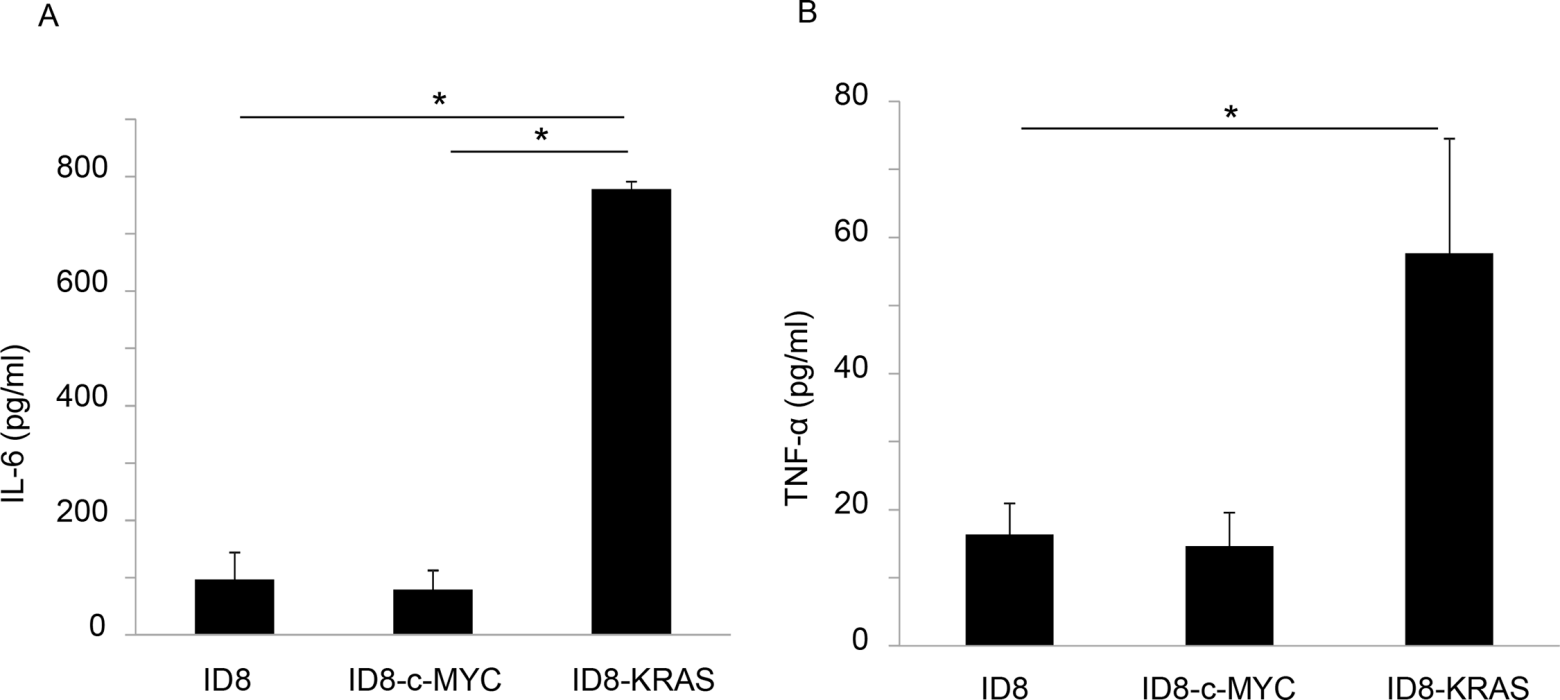
Inflammatory cytokines in ascites. ID8, ID8-c-MYC, or ID8-KRAS cells (2 × 10^6^) were intra-peritoneal injected into C57/BL6 mice. Mice were sacrificed when the body weight reached 23 g and ascites were collected (ID8, ID8-c-MYC, and ID8-KRAS: n = 10). IL-6 (A) and TNF-α (B) concentration of ascites were assessed with specific ELISA. Error bars represent the mean ± SEM. A statistical analysis was performed with the Student’s *t*-test (*P ≤ 0.05, **P ≤ 0.01).

In order to investigate the promotion of inflammation, we assessed the concentrations of inflammatory cytokines (IL-6, IL-1β, and TNF-α) in ascites obtained from mice in which BW exceeded 23 g using specific ELISA. Although IL-1β levels were not detectable in ascites, IL-6 and TNF-α were found in all groups and their concentrations were significantly higher in ID8-KRAS mice than in ID8 or ID8-c-MYC mice (Fig 3A and 3B).

### Neutrophil numbers were higher in KRAS-induced ascites

**Fig 4:**
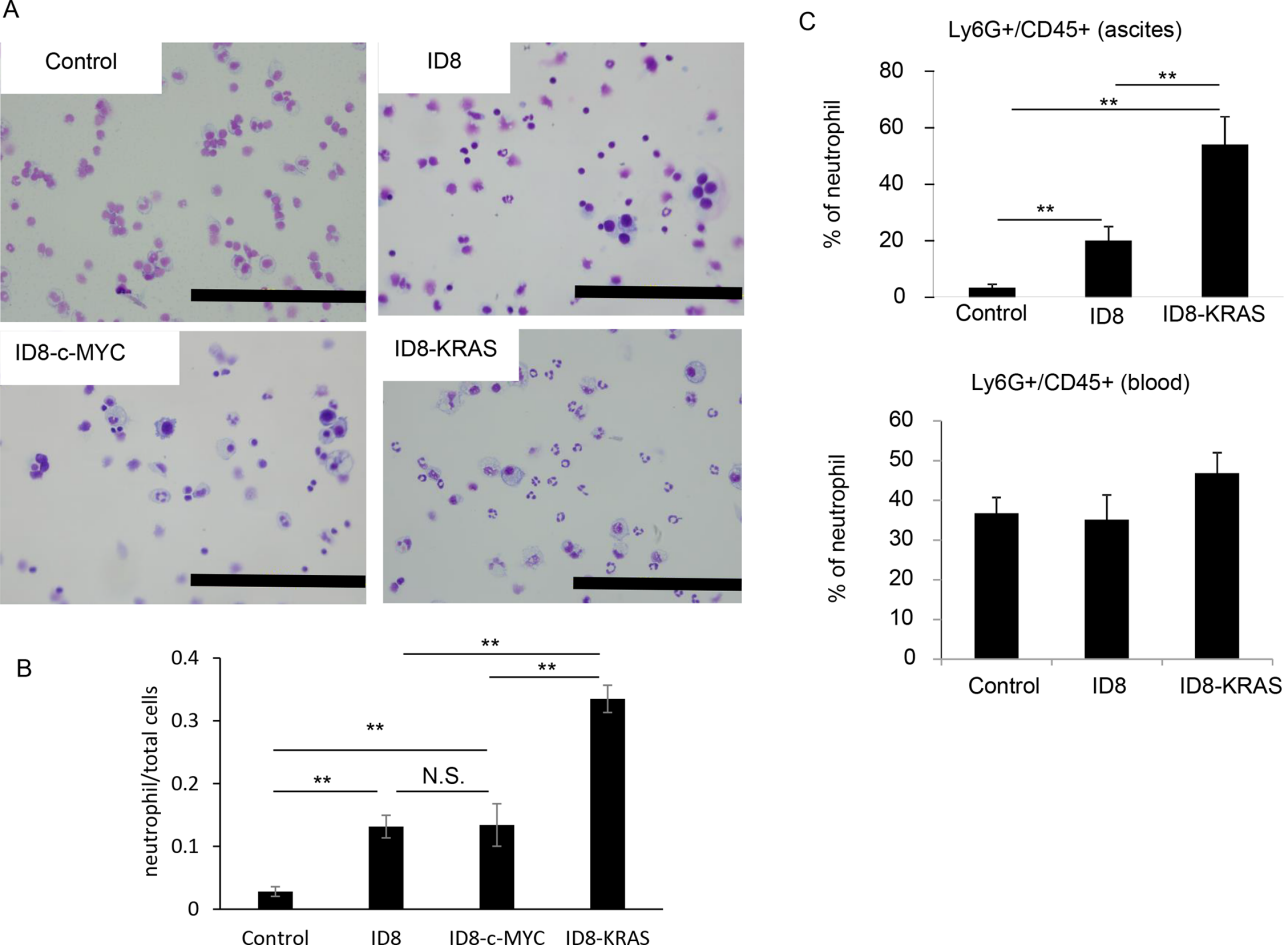
Proportion of leukocytes in ascites. ID8, ID8-c-MYC, or ID8-KRAS cells (2 × 10^6^) were intra-peritoneal injected into C57/BL6 mice. Mice were sacrificed when the body weight reached 23 g and ascites and blood were collected. Leukocytes of ascites and blood were isolated using positive selection of CD45. (A) Populations of leukocytes in the ascites were observed with Giemsa staining (2.0×10^5^ cells/mL, n = 4 in each group). Representative data was shown. Scale bars indicate 200 μm. (B) Number of neutrophils among total cells in nine high-power fields were counted in each group. Error bars represent the mean ± SEM. A statistical analysis was performed with the Student’s t-test (**P ≤ 0.01, n.s. indicates not significant.). (C) The proportion of CD45 Ly6G-positive neutrophils was assessed using flow cytometry (control: n = 4, ID8: n = 6, ID8-Kras: n = 6). Neutrophils were stained with the following antibodies: FITC-anti-mouse CD45 and APC-anti-mouse Ly6G. Error bars represent the mean ± SEM. A statistical analysis was performed with the Student’s t-test (**P ≤ 0.01).

**Fig 5:**
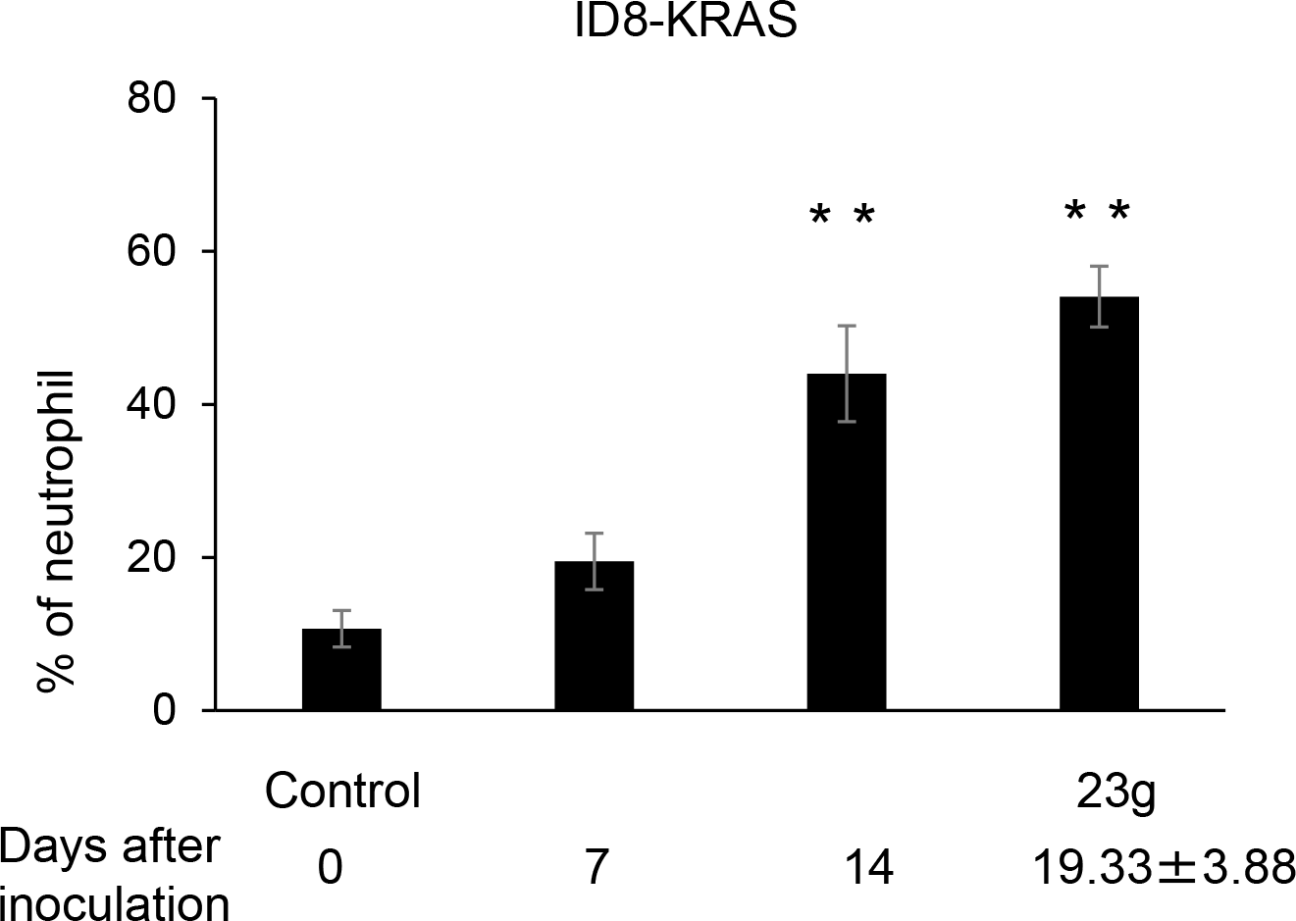
Time course analysis of neutrophil counts. ID8-KRAS cells (2 × 10^6^) were injected into the peritoneal cavities of C57/BL6 mice. ID8-KRAS mice were sacrificed on day 7(n = 6), and day 14(n = 8), and the day BW reached 23 g (n = 6). Six C57/BL6 mice were sacrificed as control. Neutrophils were stained with the following antibodies: FITC-anti-mouse CD45, APC-anti-mouse Ly6G. Dunnett's test was used to find the statistical significance compared to the control group (**P ≤ 0.01).

The inflammatory TME is characterized by the presence of host leukocytes, with macrophages, dendritic cells, mast cells, and T cells being differentially distributed [16]. Tumor-infiltrating myelocytes promote malignant tumor progression [17]. We assessed the characteristics of the population of leukocytes in ascites in each mouse group using Giemsa staining. Giemsa staining showed that neutrophils were observed markedly in ID8-KRAS-induced ascites whereas negligible in ID8- and ID8-c-MYC- ascites (Fig 4A). Further we counted the number of neutrophils in the ascites for each group (Fig 4B). The increased number of neutrophils was validated in ID8-induced ascites, which was markedly increased in ID8-KRAS-induced ascites. In order to evaluate the differential infiltration of neutrophils into ascites, we investigated the proportion of CD45 Ly6G-positive neutrophils using flow cytometry. No significant differences were observed in the number of neutrophils in blood, suggesting that KRAS did not induce neutrophilia. A time course analysis showed that, in KRAS-induced ascites, the proportion of neutrophils gradually increased from the early point of tumor progression (Fig 5).

### GM-CSF expression in ID8-KRAS mice is a possible inducer of a higher number of neutrophils

**Fig 6:**
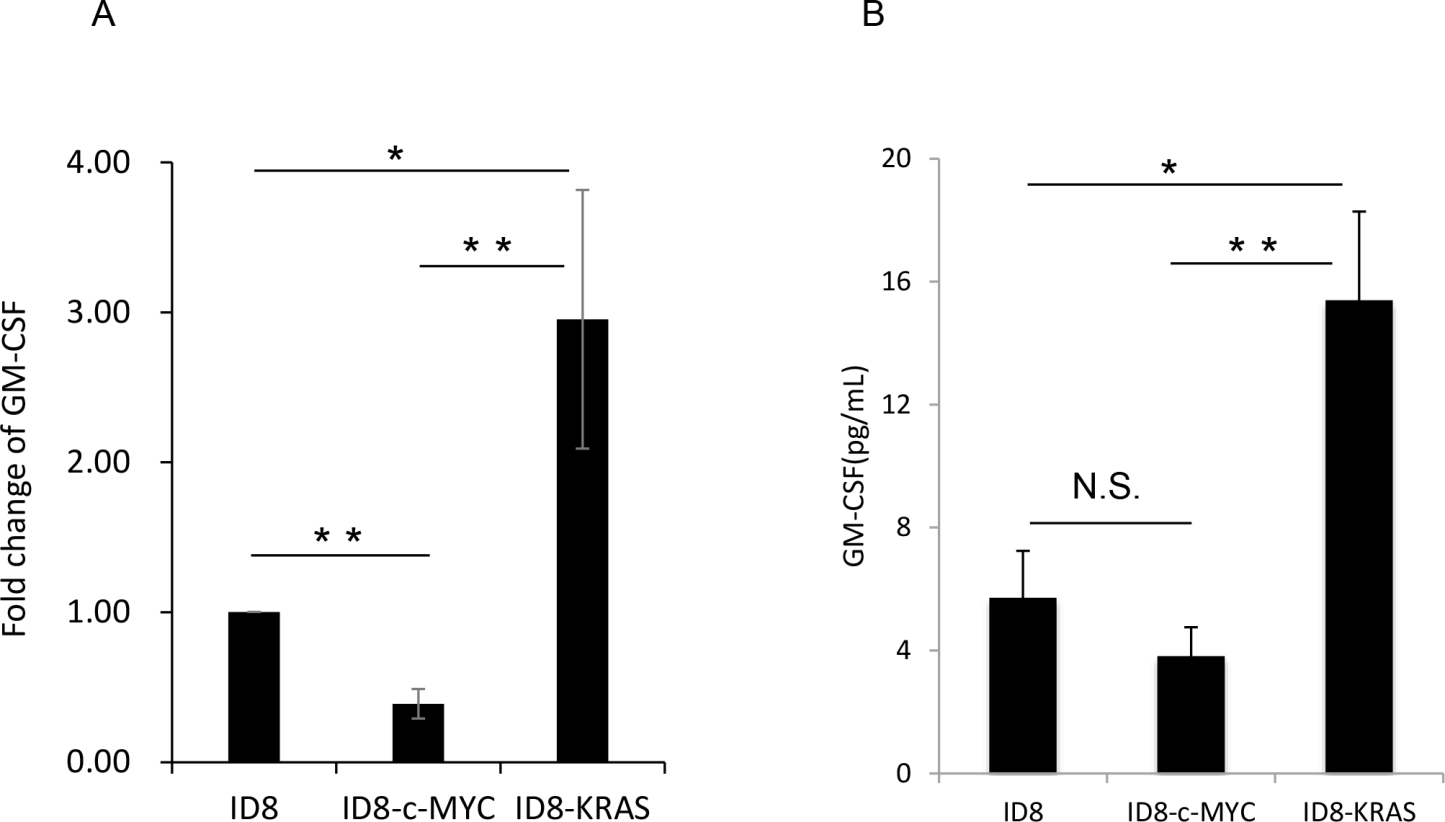
Granulocyte Macrophage Colony-stimulating Factor (GM-CSF) expression. (A) ID8, ID8-c-MYC, and ID8-KRAS cells were plated on 12-well culture plates at a concentration of 2×10^6^ cells/ml and cultured for 48 h, and total RNA was extracted followed by revers transcription. GM-CSF mRNA levels were assessed using a quantitative reverse transcription polymerase chain reaction. The expression of GM-CSF was normalized using PPIA mRNA as the internal standard. Expression levels were calculated by the comparative Ct method using PPIA as an endogenous reference gene. Data are the mean ± standard error of the mean (SEM) of three independent experiments. Data were analyzed using the Student’s t-test (*P ≤ 0.05, **P ≤ 0.01). (B) ID8, ID8-c-MYC, and ID8-KRAS cells were plated on 12-well culture plates at a concentration of 2×10^6^ cells/ml and cultured for 48 h. The concentrations of GM-CSF protein levels were assessed using specific ELISA. Error bars represent the mean ± SEM. A statistical analysis was performed with the Student’s t-test (*P ≤ 0.05, **P ≤ 0.01, n.s. indicates not significant.).

In order to investigate chemokine levels related to neutrophil migration, we performed a cytokine array of each cultured medium in order to determine the possible causes for the elevated number of neutrophils in ascites. A difference in cytokine or chemokine levels related to neutrophil recruitment or production was only observed in GM-CSF (data not shown). GM-CSF was detected only in the cultured medium of ID8-KRAS cells by cytokine array. Then we confirmed the difference in GM-CSF by RT-PCR and ELISA for GM-CSF (Fig 6A and 6B). GM-CSF expression at both mRNA and protein levels in ID8-KRAS were higher than the others. On the other hand, there was no difference in GM-CSF protein level between in ID8 and ID8-c-MYC (Fig 6B). The data suggested that GM-CSF secreted locally from ID8-KRAS cells may recruit neutrophils into the TME (the peritoneal cavity or peritoneum).

## Discussion

We herein demonstrated that c-MYC and KRAS accelerated the production of ovarian cancer-associated ascites. KRAS in particular markedly promoted ovarian cancer progression. No significant difference was observed in proliferative activity *in vitro and invitro*. VEGF concentrations were significantly high in c-MYC-induced ascites, whereas inflammatory cytokines were present at higher levels in KRAS-induced ascites and were accompanied by neutrophil recruitment. The expression of GM-CSF may be one of the possible causes for the elevated number of neutrophils in ID8-KRAS mice.

In the present study, the results of the *in vivo* investigation revealed marked differences in the progression of peritoneal carcinomatosis; however, no significant differences were noted in proliferation activity *in vitro* and *in vivo* (Fig 1D and 1E). These results suggest that these cell lines with oncogenes promote peritonitis by modulating the TME, similar to CAF or TAM, or the peritoneum to become more suitable for cancer progression.

VEGF concentrations in ascites were significantly higher in c-MYC-induced cancer than in the two other types of cancers, suggesting that angiogenesis is responsible for c-MYC-induced peritoneal cancer. A previous study showed that c-MYC together with HIF-1α induced the overexpression of VEGF, leading to tumor angiogenesis [18]. Tumor microvessels are indispensable when tumor sizes exceed 1–2 mm^3^ [19]. In the present study, difficulties were associated with evaluating vascularization in lesions due to the small size of dissemination. However, VEGF concentrations have been identified as a central regulator of the production of ascites [20]. VEGF, which has the ability to increase vascular permeability [21], may play a central role in c-MYC-induced cancer-associated ascites. VEGF production from ID8-c-MYC cell was not higher than that from ID8 or ID8-KRAS cell (S1 and S2 Figs). We cannot explain from our present data why concentration of VEGF in ascites of ID8-c-MYC group was higher compared with the other groups. It may be one of the reasonable explanations that ID8-c-MYC cell interacts with peritoneal components which secondarily promote VEGF secretion, although the components remains unknown in our study.

In contrast to c-MYC-induced ascites, VEGF concentrations were similar in KRAS- and ID8-induced ascites. However, the most unusual characteristic observed was enhanced inflammation. Inflammatory cytokine levels such as those of IL-6 and TNF-α were significantly increased in KRAS-induced ascites, suggesting that inflammation plays a central role in KRAS-induced cancer progression. Another study demonstrated that RAS-induced IL-8 expression was required for tumor-associated inflammation and neovascularization [22]. KRAS-induced inflammation has been extensively examined in lung cancer [23]. RAS is considered to promote cancer progression by sustaining proliferation, metabolic reprogramming, anti-apoptosis, and remodeling of the TME [24, 25]. RAS followed by the activation of RAS-GTP and PI3K/AKT signaling may up-regulate NF-κB activity and lead to an inflammatory microenvironment [4]. In order to clarify how KRAS induces inflammatory signaling, we assessed IL-6 mRNA expression in each cell line, however IL-6 was almost undetectable in any cell lines (data not shown). Therefore we considered that KRAS signaling somehow interacts with TME and inflammatory cytokines might be secreted from components of TME. Among them neutrophils are one of the famous inflammatory cells which can produce IL-6, therefore the increased number of neutrophils might be one reason for the higher concentration of IL-6 in KRAS-induced ascites. The relationship between inflammation and cancer has been a well-studied issue. In the course of cancer progression, cancer cells and TME interact with each other, resulting in chronic inflammation [26]. CAF or TAM secretes IL-6, which induces cancer EMT (epithelial-mesenchymal transition) or an immunosuppressive condition, thereby contributing to cancer progression [7, 8]. In ovarian cancers, the induction of IL-6 by the co-existence of an ARID1A-PIK3CA mutation has been shown to promote ovarian clear cell carcinoma [6]. Another study proposed that the selective targeting of IL-6 trans-signaling *in vivo* reduces the formation of ascites [27, 28]. Collectively, these findings suggest that enhanced inflammation plays a role in the progression of KRAS-induced ovarian cancer.

Another characteristic of the KRAS-induced TME is an increased number of neutrophils in ascites. Accumulating evidence from clinical trials has revealed that neutrophilia or a high neutrophil-lymphocyte ratio indicates a poor prognosis [29, 30]. However, the mechanisms responsible for increasing neutrophil numbers have not yet been elucidated in detail. Our results suggest that regardless of the amount of ascites or formation of dissemination, KRAS increases the number of recruited neutrophils. We also propose that oncogenes play a role in increasing the number of neutrophils. Previous studies demonstrated that lung cancer with a KRAS mutation showed increased numbers of neutrophils through the expression of CXCL-1 and CXCL-2 [26]. In an attempt to determine how KRAS induces increases in neutrophil numbers, we assessed chemokine levels in ascites or cultured complete media; however, no significant differences were observed in chemokine levels among the 3 groups. We then focused on the cultured complete medium and performed a cytokine array. Among the cytokines and chemokines related to neutrophil production or recruitment on the panel, only GM-CSF was detected in ID8-KRAS cultured medium. GM-CSF originally received attention as a hematopoietic growth factor that stimulates the production of neutrophils and macrophages. Recent findings have shown that in addition to the stimulation of granulocyte production, GM-CSF has the ability to recruit neutrophils from the microvasculature, thereby inducing extravascular migration *in vivo* [31] and increasing the functional life span of neutrophils [32]. In our model, GM-CSF in the ascites was not detected by ELISA. The cancer-related recruitment of neutrophils might occur at the early phase of cancer dissemination into the peritoneal cavity. Otherwise, GM-CSF secreted from ID8-KRAS cells might act on neutrophils locally in the disseminated lesions recruiting them from circulation, although GM-CSF concentration was diluted by massive ascites. Our data suggest that KRAS-induced GM-CSF expression was one of the causes for the increased number of neutrophils in the TME.

This study has several limitations. Firstly, the oncogenes transduced into ID8 are derived from human beings. Therefore there is a possibility that unpredictable reactions including immune responses to foreign antigen would be induced especially in vivo. The change of TME in immune-deficient mice is considered to be different from immunocompetent one. Therefore we chose immunocompetent mice to observe the changes of TME. Further investigation by inducing mouse active form of mutations in ID8 is needed to precisely examine the effects of pure oncogene on the TME. However, the lack of studies on murine KRAS or c-MYC hampers insert of mutations corresponding to human oncogenes into the mouse genes. Secondly, in the present study, we did not assess the therapeutic effects of anti-inflammatory drugs or anti-angiogenic drugs on KRAS- or c-MYC-induced peritonitis.

We herein propose the generation of more aggressive peritoneal cancer mouse models by transducing oncogenes into ID8. One of the mechanisms used by oncogenes to promote ovarian cancer progression may be modulation of the TME. The relationship between oncogenes and their specific TME modulations may contribute to a clearer understanding of the mechanisms underlying cancer progression. Therefore, further investigations are needed in order to identify biomarkers for suitable therapeutics. These findings will provide support for therapeutics corresponding to driver oncogenes being more effective as precision medicine for ovarian cancer.

## Supporting Information

S1 Fig VEGF expression levels *in vitro*. (TIFF)

S2 Fig VEGF expression in disseminations. (TIFF)

S1 Table Primer pairs and probe numbers. (TIF)
