# Supplementary figures and images for: Modification of the Tumor Microenvironment in KRAS or c-MYC-Induced Ovarian Cancer-Associated Peritonitis
Source: PLoS One. 2016 Aug 2;11(8):e0160330. doi: 10.1371/journal.pone.0160330 (PMC4970724; doi:10.1371/journal.pone.0160330)

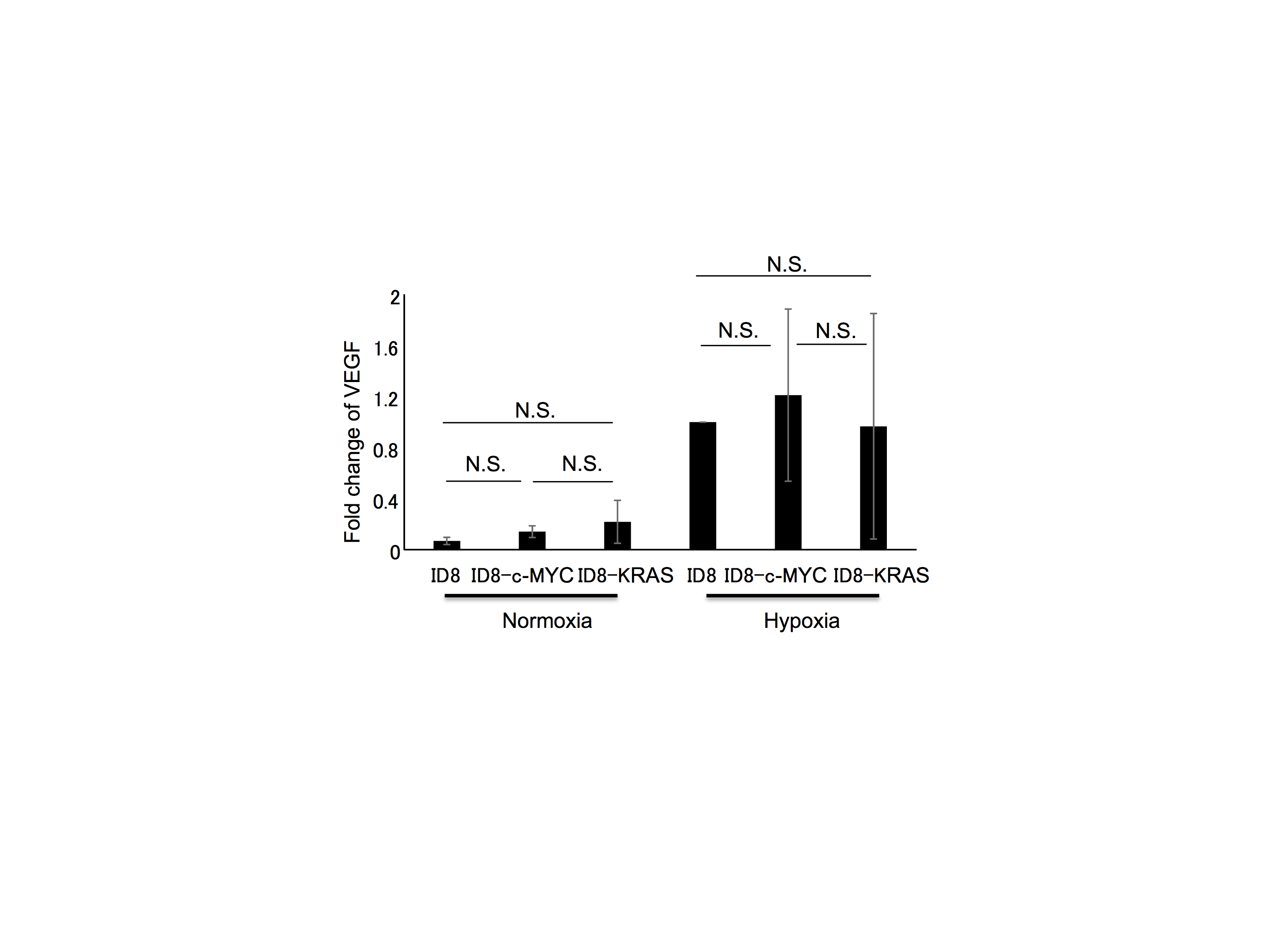

Supplement: S1 Fig — (TIFF) [file pone.0160330.s001.tiff]

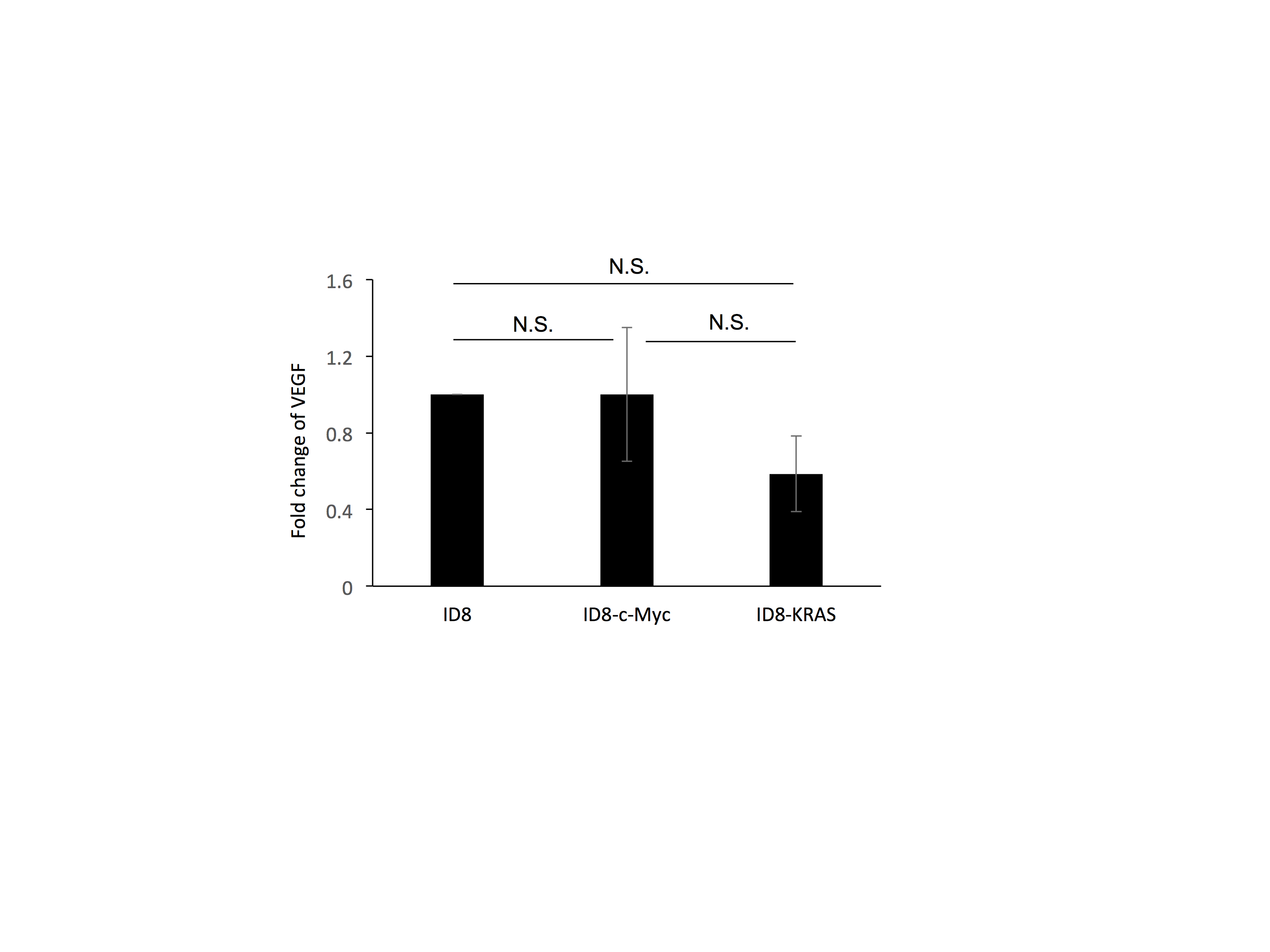

Supplement: S2 Fig — (TIFF) [file pone.0160330.s002.tiff]

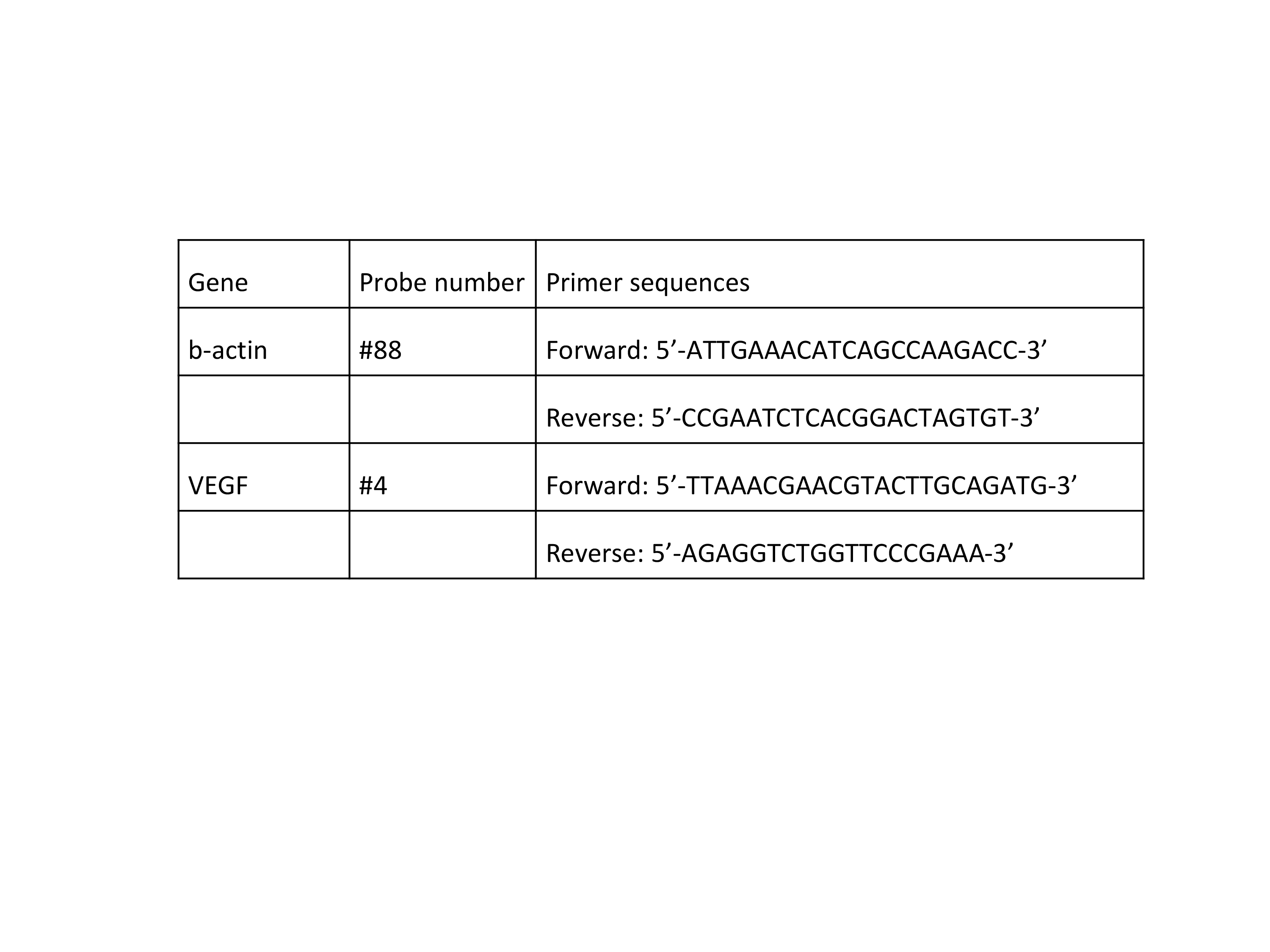

Supplement: S1 Table — (TIF) [file pone.0160330.s003.tif]
